# Optimizing Anti-Viral Vaccine Responses: Input from a Non-Specialist

**DOI:** 10.3390/antibiotics9050255

**Published:** 2020-05-15

**Authors:** Philip Serwer

**Affiliations:** Department of Biochemistry and Structural Biology, The University of Texas Health Center, San Antonio, TX 78229-3900, USA; serwer@uthscsa.edu; Tel.: +1-210-567-3765

**Keywords:** bacteriophage, coronavirus, directed evolution, emerging viral pathogen, vaccine

## Abstract

Recently, the research community has had a real-world look at reasons for improving vaccine responses to emerging RNA viruses. Here, a vaccine non-specialist suggests how this might be done. I propose two alternative options and compare the primary alternative option with current practice. The basis of comparison is feasibility in achieving what we need: a safe, mass-produced, emerging virus-targeted vaccine on 2–4 week notice. The primary option is the following. (1) Start with a platform based on live viruses that infect bacteria, but not humans (bacteriophages, or phages). (2) Isolate phages (to be called pathogen homologs) that resemble and provide antigenic context for membrane-covered, pathogenic RNA viruses; coronavirus-phage homologs will probably be found if the search is correctly done. (3) Upon isolating a viral pathogen, evolve its phage homolog to bind antibodies neutralizing for the viral pathogen. Vaccinate with the evolved phage homolog by generating a local, non-hazardous infection with the phage host and then curing the infection by propagating the phage in the artificially infecting bacterial host. I discuss how this alternative option has the potential to provide what is needed after appropriate platforms are built.

## 1. Introduction

When a distant goal is to be reached, strategies of two types typically evolve. (1) Establish the overall objective and then establish matching subsidiary objectives, with little regard for how difficult the subsidiary objectives are. The point is that, if the subsidiary objectives are reached, then the overall objective is reached. (2) Establish readily doable subsidiary objectives and delay concern about fitting them together to reach the overall objective. Some enterprises begin with a type 1 strategy and are then infiltrated by what might be called a creeping type 2 strategy. One reason is that a type 2 strategy has the most immediate appeal, especially when setting fundable goals and trying to maintain organizational status quo.

An example of a creeping type 2 strategy is provided by the Ranger program to photograph the moon in the early days of the US space program. The first six missions failed [1]. Although the details differed for the different failures [1], one can summarize the failures as the products of deficient systems integration, unrealistic testing conditions and haste. The deficiency in systems integration is basically a synonym for a creeping type 2 strategy. Haste and lack of real-world orientation are corollary to a type 2 strategy. Better adherence to a type 1 strategy yielded more success with the initially-believed-beyond-reach, 9 year targeted Apollo program [2,3]; people walked on the moon ahead of schedule [3,4]. Closer to the current topic is a non-specialist critique of research on curing metastatic cancer. In brief, this discussion outlines a creeping type 2 strategy in that field [5].

The central point here is that the situation is analogous for developing vaccines for pandemic viruses. That is to say, as discussed in more detail below, vaccine-producing efforts have the highest chance of success via disciplined use of a type 1 strategy. More than one type 1 strategy option exists for reaching the overall objective. The overall objective is a safe, mass-produced, emerging virus-targeted vaccine on 2–4 week notice. Here, I describe details of the subsidiary objectives of my primary type 1 strategy option. All subsidiary objectives are based on platforms that are (1) rapidly, but without haste, used after being built and (2) initially, at least, difficult to build and dependent on highly trained, highly motivated personnel. I compare the prospects with the prospects of current work in other directions. When viewed from the perspective of the overall objective, most to all of the latter are creeping type 2 strategy options. I also present an alternative type 1 strategy option.

For the main type 1 strategy option, the following are the initial subsidiary objectives for the overall objective. (1) Start with a platform based on live viruses that infect bacteria, but not humans (bacteriophages, or phages [6,7]). (2) Isolate phages (to be called pathogen homologs) that resemble and provide antigenic context for membrane-covered, pathogenic RNA viruses; coronavirus-phage homologs might be found if the search is correctly done. (3) Upon isolating a pathogenic virus, evolve its phage homolog to bind neutralizing antibodies to the pathogenic virus. (4) Vaccinate with the evolved phage homolog by generating a local, non-hazardous infection with the phage host and then curing the infection by propagating the phage in the artificially infecting host.

## 2. Large-Scale Vaccine Production and Use

The last subsidiary objective of any strategy is mass vaccine production. If this subsidiary objective is unachievable, not much point exists to working on the others. Live vaccines are the most easily adapted to meet this requirement because their constituents propagate post-vaccination. Smallpox vaccine [8] and the Sabin [9] and Koprowski [10] polio vaccines are examples; the Salk inactivated virus vaccine was the first vaccine for polio [11]. Indeed, most currently approved anti-viral vaccines are either attenuated, live virus vaccines or inactivated virus vaccines [12,13,14,15]. In the case of phages, a single preparation typically has over 10^13^ phage particles (sometimes over 10^14^) when the phages have been propagated, concentrated and purified from a 15 L lysate. This is more than enough to vaccinate everyone in the world with a 1000 phage dose, if the phages can be made to propagate post-vaccination. Making a 15 L lysate takes approximately one day. Bulk-purifying phages by ultracentrifugation takes approximately two days.

To achieve post-vaccination phage propagation, we have to give the phages a bacterial host. One way to do this is to start an intra-dermal infection with the bacterial host. Then, cure the infection by infecting the bacterial host with the antigenic phage. This is upside-down phage therapy so to speak. The phages will multiply until either the bacteria are gone or until immune systems prevent further multiplication. Thus, the characteristics of the bacterial host are key components of this platform. The host must not itself become a problematic pathogen, yet it must continue to propagate enough to yield enough phages to stimulate innate and adaptive immune systems to produce immunity to the phage and, by homology, immunity to the virus that is causing the problem in the first place.

Indeed, this proposed procedure is not all that far from the procedure of a smallpox vaccination and might leave a similar scar. Some of us can still see scars from smallpox vaccination. The main difference is that the replicating phage antigen needs externally supplied host cells in order to produce immunity.

Without going into details concerning live vaccine production via eukaryotic viruses, I think it reasonable to assume that eukaryotic virus production is more difficult, more expensive and less rapid than the production of phages. Further, there are fewer safety concerns with phages.

## 3. Evolving Phages to Bind Antibodies that Neutralize a Viral Pathogen

To understand the principle of how this type of evolution might be achieved, I use the following analogy. Suppose one wanted to maximize the speed of the non-mechanized elevation of a person to a ledge 10 feet high. Jumping that high is out of the question, no matter how many tries are made. More realistically, one would make and use a series of less incremental ledges, steps so to speak. The situation with phages is analogous, as we found when evolving phage T3 to propagate in 0.9 M NaCl. This had to be done in steps [16]. Parenthetically, this NaCl concentration is so high that, in Petri plates, our only indication of host growth was the observation of plaques of our step-evolved phage. This example emphasizes the basic principle. It is not intended to indicate the complexity of what is proposed below.

Thus, the basic idea is to build the foundation by step-evolving phages to react with the neutralizing antibodies of the target pathogen. I assume here that the antibodies are already in hand. Meeting the overall objective implies that some of the step evolving has to be done before the pathogen exists. That, in turn, can be done by antibody-selecting phage homologs for each of the multiple known and potential pathogens isolated from the environment. For example, an antibody-selected SARS-CoV-1 phage homolog might serve as the starting point for an antibody-selected SARS-CoV-2 phage homolog. That is to say, some of the inter-pandemic activity would be devoted to improving vaccines to current pathogens. Recommendations to do this have already been made, in the context of other vaccine-generating options [14].

Several procedures exist for performing the antibody-selection of phages. Affinity, antigen-loaded columns are already used via chromatography to select antibody-displaying phages for antibodies with increased affinity to antigen [17,18]. Using this procedure, while reversing the role of antibody and antigen (antibody bound to the column), is one way to antibody-select phages. An independent approach is indicated by the fact that antibody-generated phage multimers are observed as discrete, more origin-proximal-than-monomeric-phage bands after reacting phages with antiserum and performing native gel electrophoresis [19]. Selecting phages near or at the origin is an alternative selection of antibody-reactive phage. The same is the case for phages that are in the void volume after molecular sieve chromatography. Selecting rapidly sedimenting phages after rate zonal centrifugation in a sucrose density gradient is yet another procedure.

Then arises the question of which phage to use at the beginning. In theory, any stable phage could be used as a starting point. Time to objective might be decreased by using a phage displaying a peptide from the spike protein (or other protruding protein) of a potential target virus. However, current efforts to human-engineer improved antigens for anti-RNA virus vaccines have shown that neutralizing antibodies typically react with viral proteins that are in states that are context dependent and unstable [12,13,15,20]. The context includes assembly with other viral proteins, which results in the use of artificial scaffolds in structural vaccinology [12]. Much work has been devoted to stabilizing protein antigens in “appropriate” states and masking epitopes that interfere with the epitope that reacts with neutralizing antibody [12,13,15,20]. RNA viruses have evolved sophistication to the point that they make most accessible the epitopes that change the most rapidly [20]. The context also includes embedding in a lipid bilayer membrane [12,13,14,15]. The context dependence of neutralizing antibody antigenicity leads to the conclusion that, in practice, the best starting point is a phage structurally homologous to the known or anticipated pathogenic virus.

I take the liberty of responding here to the obvious objection that no membrane-covered, single-stranded RNA phage has ever been isolated [21] and that the pandemic viruses include influenza, Zika-type and coronaviruses, all in this category. This non-isolation does not imply non-existence. Membrane-covered, double-stranded RNA phages have been isolated [21]. Already shown is that membrane-coated DNA phages are dramatically under-isolated with the current most popular isolation procedures. Chloroform treatment is a major culprit [22]. The time is now to make the appropriate isolation attempts. I note that some propagating, bacteriolytic particles are, structurally, far from the phages isolated in the past; an example is phage 904phi4-5 in [23].

The underlying thesis for this section is that genetic selection is the most reliable way to achieve the systems engineering needed to achieve appropriate antigens for needed vaccines. When performed with phages, genetic selection is also rapid to the point that human engineering, even if enough was known to do it reliably, would probably not be as rapid.

## 4. Isolating Phage Homologs to Disease-Causing Viruses

My preferred procedural advance for rapidly and unbiasedly isolating phages has already been described [24,25]. In brief, the phage isolation techniques include both initial isolation and subsequent propagation in a gel. All transfers are done with a needle. No liquid culture is involved. One advantage of a procedure of this type is that dry environmental samples (often in a powdered state) are used, thereby increasing the probability that the phages isolated can be dried for transport. Presumably, further development of these procedures is possible.

Organization and inter-laboratory coordination will be a key feature of any effort, whatever procedures are used for phage isolation. The reason is that the phage collection will be large enough so that computerization will be needed for access to both data and the phages themselves. Organization begins with a naming system that makes impossible the giving of the same name to two different phages [24,25].

## 5. Safety

Any therapeutic use of phages begins with two advantages in the area of safety. (1) Although phage uptake into human cells occurs, no evidence exists that phages replicate in human cells [26,27,28]. For example, phages use transcription and translation signals that are different from those of eukaryotic cells. (2) The experience with phage therapy of infectious disease is that the phage particles themselves are never found to have negative effects in humans [29,30]. Of course, that does not indicate zero danger. Perhaps the greatest danger is the induction of non-neutralizing antibodies that aggravate an infection. This is one explanation of vaccine aggravation of dengue fever, a phenomenon preferentially encountered with young recipients of dengue fever vaccine [31,32]. However, selection against induction of non-neutralizing antibodies occurs when the phage is selected for binding to neutralizing antibody.

One cannot be so sanguine about any vaccine that has either eukaryotic RNA or eukaryotic DNA with transcription and translation signals. DNA vaccines are the more serious concern because of possible chromosome insertions [13]. In the case of an un-encapsulated RNA vaccine, a secondary concern is destruction of the vaccine by RNases [13]. Assuming that eukaryotic DNA and RNA vaccines will always have to be safety tested, they are not likely to meet the 2–4 week constraint that is imposed by a novel, rapidly spreading pandemic virus. Many years are likely to be required to test for cancer-generating chromosomal alterations. A non-specialist observer reasonably concludes that DNA and RNA vaccines, when viewed in the context of our overall objective, are examples of type 2 strategy options. Furthermore, in spite of a ~27 year history [33], no DNA vaccine has ever been FDA approved for use with humans [13,34]. Pathogen homolog RNA phage vaccines, on the other hand, would have an a priori and reasonable projection (not certainty) of safety. Even some DNA phage vaccines might eventually be placed in this category.

The importance of this latter characteristic becomes apparent when one intellectually removes the blinders that human emotion places on us. Specifically, SARS-CoV-2 is not as deadly as SARS-CoV-1. The problem is that SARS-CoV-2 transmits more actively and does so on inanimate surfaces, although I have not yet seen a claim that SARS-CoV-2 can transmit on dust particles. Can a virus transmit even more actively than SARS-CoV-2? A combination thought/real-world experiment suggests that this can happen. First, in the real world, one of the best sources of phages is dry soil around horse watering troughs (our circumstantial observations). This soil typically resembles a powder and would be easily dispersed as dust in the atmosphere by wind. That is to say, some phages are reasonably assumed to survive on dust particles. If so, the thought part of this experiment tells us that eukaryotic viruses can also evolve to survive on dust particles. Similar, more dramatic dust-generating effects occur in deserts. We were apparently lucky that the MERS coronavirus [35] did not create greater problems than it did via transmission on sand particles, given that it originated in Saudi Arabia.

Can more active transmission be accompanied by higher virulence? I will not make a projection. However, we have to be prepared for this possibility. Either way, I think that the case for vaccine development speed, and therefore the case for vaccines with a priori, rational expectation of safety, needs no further justification. I do not speculate here on what the details should be for safety standards.

## 6. Speed

Intuitively obvious is that no current, platform-ready technology can meet the time constraint in the overall objective used here. Otherwise, we would already have a vaccine and we would not be told that we have to wait over a year for one. My use of this time constraint is closely connected to the real world of both mass fatalities and economic collapse—both of which are now beginning and can be compounded by criminal misuse of SARS-CoV-2. That is to say, realistic conditions are not being currently used for testing vaccine strategies. The analogy with the early stages of the Ranger program is too close to miss.

Thus, I have trouble conceiving a good reason why a call for new options has not been made. I am reminded of two folk sayings (I am not sure who are the originators). (1) “Most people would rather fail conventionally than succeed unconventionally.” (2) When the tide goes out, you discover who is swimming naked.” The tide is going out.

## 7. Perspective and an Alternative

The preferred option for anti-viral vaccine response depends, to some extent, on the history and agenda of the responder. The option proposed above is obviously a product of the author’s history. Indeed, the phage isolation aspect overlaps the author’s previous proposals for resolving current crises in the therapy of multi-drug-resistant bacterial infections [36] and metastatic cancer [37]. Investigation of natural phage bioengineering has produced a framework for understanding and curing neurodegenerative diseases [38]. The underlying thesis is the following: make use of the systems engineering of perhaps two billion years of phage evolution.

Given that the platforms presented here for the above type 1 strategy option are not (and may never be) built, I present an alternative to this option. The alternative option is a live eukaryotic virus vaccine of atypical type. This option is designed for the time constraint of the overall objective, 2–4 weeks. Given that eukaryotic viruses have doubling times much greater than those of phages (2–5 min for typical coliphages), meeting this objective implies that a live virus vaccine has to be already present in the environment.

That this presence is a realistic expectation was shown by Pasteur’s demonstration of the absence, for all practical purposes, of spontaneous generation of microbes [39]. Thus, any emerging pathogenic virus has ancestors. Given the emerging pathogen character, most ancestors do not have the pathogenicity of the current pathogen. However, at least some ancestors are likely to have sufficient antigenic overlap to act as live vaccines. That use of such ancestor vaccines can work has been shown via finding that porcine respiratory coronavirus infection of pigs immunizes naturally against the more dangerous transmissible gastroenteritis coronavirus [40]. Further, the original smallpox vaccine was also of this type [8].

Thus, the first platform for this alternate type 1 strategy option is a library of live viruses in each of the classes that also contain viral pathogens. Recommendation to build this platform has already been made for other reasons [14].

The second platform is enough interpreted data so that both pathogenicity and antigenicity of any potential live vaccine strain can be predicted from the base sequence of its genome. This platform is not here at present and is faced with significant difficulties. For example, even though the SARS-CoV-1 spike protein (i.e., the protein that makes the initial contact with a cellular receptor) is structurally homologous to the SARS-CoV-2 spike protein, neutralizing antibodies to the SARS-CoV-1 spike protein do not react with the SARS-CoV-2 spike protein [41]. Further, one might find that predictions made always had some level of uncertainty. One of the reasons for building/sequencing/interpreting an extensive library of phages is to assist interpretation of the sequences of eukaryotes and their viruses.

The decision to make use of fast-tracked live vaccines would presumably depend on the state of the pandemic being faced. As a thought experiment, imagine that (1) you are 80 years old today, (2) a fast-tracked live-virus vaccine has been developed for SARS-CoV-2 and (3) a reasonable projection is that you have a 1/100 chance of being seriously ill from the vaccine. The bias toward or against vaccination would depend on the projection for how socio-political measures will go in the future. As the economy progressively pressures the easing of these measures, I suspect that the bias for most people will progressively become toward vaccination.

The real world of “needed research” can be (and, in my opinion, is) different from the world of “fundable research”. Currently, the latter typically implies a type 2 strategy, often with tenuous links to the overall objective. I am talking about both government and private foundation funding. I am also talking about bait-and-switch tactics implicit in some funding announcements. Specifically, an announcement will ask for something novel, paradigm changing, realistic and needed, while embedding a requirement that precludes some or all of the above. If this requirement is not stated, the process can be reasonably called corrupted. The experience with generating polio virus vaccines [42] suggests that non-specialist scientific influence will be needed to manage the conditions described in this paragraph. The “silver lining” is that the recent crisis might help make improvements in research on therapies for diseases other than pandemic virus infections.

## 8. A Note for Doubters

The following examples will, perhaps, convince those who are doubters of the value of a type 1 strategy. Paul Ehrlich used a type 1 strategy to plough through compounds until he found the first usable antibiotic, Salvarsan (reviewed in [43]). Schatz and Waksman did the same during the discovery of streptomycin [44]. Early anti-cancer drug discovery was a repeat performance (reviewed in [5]).

Furthermore, the current situation with SARS-CoV-2 has been described as analogous to war [45]. Of course, in this war, everyone is on the same side, whether or not apparent. Military strategy competency credentials aside for the moment, I could not help noticing that a type 1 strategy appears to have been the more successful strategy in war. One illustrative example is the multi-easy victory, but lack-of-a-central objective and ultimately losing, type 2 strategy of Hannibal in the second Punic War [46]. (This example was recommended to me by an in-house reader of this manuscript, Richard Ludueña.) Given its historical closeness, I also compare the poorly coordinated, low-training, take-the-easy-path, ultimately disastrous type 2 strategy of the US at the beginning of the Battle of the Bulge with the initially-thought-impossible-to-execute, focused, rapid, highly coordinated, successful type 1 strategy used by the highly trained, highly motivated US third army at the end of the Battle of the Bulge [47]. Let us hope that, in the contest with SARS-CoV-2, the outcome for people resembles the latter US outcome.

## References

[1] Hall R.C. (1977). Lunar Impact: A History of Project Ranger.

[2] Matranga G.J., Ottinger C.W., Jarvis C.R., Gelzer D.C. (2005). Unconventional, Contrary, and Ugly: The Lunar Landing Research Vehicle (The NASA History Series).

[3] Kranz G. (2000). Failure Is Not an Option: Mission Control: From Mercury to Apollo 13 and Beyond.

[4] Berg J. (2019). A child of Apollo. Science.

[5] Leaf C. (2013). The Truth in Small Doses: Why We’re Losing the War on Cancer-and How to Win It.

[6] Hyman P. (2019). Phages for phage therapy: Isolation, characterization, and host range breadth. Pharmaceuticals.

[7] Hatfull G.F. (2008). Bacteriophage genomics. Curr. Opin. Microbiol..

[8] Sánchez-Sampedro L., Perdiguero B., Mejías-Pérez E., García-Arriaza J., Di Pilato M., Esteban M. (2015). The evolution of poxvirus vaccines. Viruses.

[9] Cáceres V.M., Sutter R.W. (2001). Sabin monovalent oral polio vaccines: Review of past experiences and their potential use after polio eradication. Clin. Infect. Dis..

[10] Koprowski H., Jervis G.A., Norton T.W. (1954). Administration of an attenuated type 1 poliomyelitis virus to human subjects. Proc. Soc. Exp. Biol. Med..

[11] Juskewitch J.E., Tapia C.J., Windebank A.J. (2010). Lessons from the Salk polio vaccine: Methods for and risks of rapid translation. Clin. Transl. Sci..

[12] Loomis R.J., Johnson P.R. (2015). Emerging vaccine technologies. Vaccines.

[13] Rauch S., Jasny E., Schmidt K.E., Petsch B. (2018). New vaccine technologies to combat outbreak situations. Front. Immunol..

[14] Graham B.S., Sullivan N.J. (2018). Emerging viral diseases from a vaccinology perspective: Preparing for the next pandemic. Nat. Immunol..

[15] Anasir M.I., Poh C.L. (2019). Structural vaccinology for viral vaccine design. Front. Microbiol..

[16] Serwer P., Wright E.T., Liu Z., Jiang W. (2014). Length quantization of DNA partially expelled from heads of a bacteriophage T3 mutant. Virology.

[17] Ubah O., Palliyil S. (2017). Monoclonal antibodies and antibody like fragments derived from immunised phage display libraries. Adv. Exp. Med. Biol..

[18] Shim H. (2017). Antibody phage display. Adv. Exp. Med. Biol..

[19] Serwer P., Hayes S.J. (1982). Detection of bacteriophage-antibody complexes by agarose gel electrophoresis. Electrophoresis.

[20] Tobin G.J., Trujillo J.D., Bushnell R.V., Lin G., Chaudhuri A.R., Long J., Barrera J., Pena L., Grubman M.J., Nara P.L. (2008). Deceptive imprinting and immune refocusing in vaccine design. Vaccine.

[21] Mäntynen S., Sundberg L.R., Oksanen H.M., Poranen M.M. (2019). Half a century of research on membrane-containing bacteriophages: Bringing new concepts to modern virology. Viruses.

[22] Kauffman K., Hussain F., Yang J., Arevalo P., Brown J.M., Chang W.K., VanInsberghe D., Elsherbin J., Sharma R.S., Cutler M.B. (2018). A major lineage of non-tailed dsDNA viruses as unrecognized killers of marine bacteria. Nature.

[23] Serwer P., Wang H. (2006). Single-particle light microscopy of bacteriophages. J. Nanosci. Nanotechnol..

[24] Serwer P., Hayes S.J., Zaman S., Lieman K., Rolando M., Hardies S.C. (2004). Improved isolation of undersampled bacteriophages: Finding of distant terminase genes. Virology.

[25] Serwer P., Hayes S.J., Thomas J.A., Hardies S.C. (2007). Propagating the missing bacteriophages: A large bacteriophage in a new class. Virol. J..

[26] Lehti T.A., Pajunen M.I., Skog M.S., Finne J. (2017). Internalization of a polysialic acid-binding *Escherichia coli* bacteriophage into eukaryotic neuroblastoma cells. Nat. Commun..

[27] Barr J.J. (2017). A bacteriophages journey through the human body. Immunol. Rev..

[28] Dąbrowska K. (2019). Phage therapy: What factors shape phage pharmacokinetics and bioavailability? Systematic and critical review. Med. Res. Rev..

[29] Górski A., Międzybrodzki R., Łobocka M., Głowacka-Rutkowska A., Bednarek A., Borysowski J., Jończyk-Matysiak E., Łusiak-Szelachowska M., Weber-Dąbrowska B., Bagińska N. (2018). Phage therapy: What have we learned?. Viruses.

[30] Nikolich M.P., Filippov A.A. (2020). Bacteriophage therapy: Developments and directions. Antibiotics.

[31] Cohen J. (2020). New dengue vaccine performs well in large trial, but safety remains key concern. Science.

[32] Thomas S.J., Yoon I.K. (2019). A review of Dengvaxia^®^: Development to deployment. Hum. Vac. Immunother..

[33] Alarcon J.B., Waine G.W., McManus D.P. (1999). DNA vaccines: Technology and application as anti-parasite and anti-microbial agents. Adv. Parasitol..

[34] Hobernik D., Bros M. (2018). DNA vaccines-How far from clinical use?. Int. J. Mol. Sci..

[35] Mackay I.M., Arden K.E. (2015). MERS coronavirus: Diagnostics, epidemiology and transmission. Virol. J..

[36] Serwer P. (2017). Restoring logic and data to phage-cures for infectious disease. AIMS Microbiol..

[37] Serwer P., Wright E.T., Gonzales C.B., Khalil I. (2020). Phage capsids as gated, long-persistence, uniform drug delivery vehicles. Current and Future Aspects of Nanomedicine.

[38] Serwer P., Hunter B., Wright E.T. (2020). Electron microscopy of in-plaque phage T3 assembly: Proposed analogs of neurodegenerative disease triggers. Pharmaceuticals.

[39] Dubos R.J. (1950). Louis Pasteur, Free Lance of Science.

[40] Vlasova A.N., Wang Q., Jung K., Langel S.N., Malik Y.S., Saif L.J. (2020). Porcine coronaviruses. Emerg. Transbound. Anim. Viruses.

[41] Wrapp D., Wang N., Corbett K.S., Goldsmith J.A., Hsieh C.L., Abiona O., Graham B.S., McLellan J.S. (2020). Cryo-EM structure of the 2019-nCoV spike in the prefusion conformation. Science.

[42] Rose D.W. (2016). Friends and Partners: The Legacy of Franklin D. Roosevelt and Basil O’Connor in the History of Polio.

[43] Bosch F., Rosich L. (2008). The contributions of Paul Ehrlich to pharmacology: A tribute on the occasion of the centenary of his Nobel Prize. Pharmacology.

[44] Schatz A., Waksman S.A. (1944). Effect of streptomycin upon *Mycobacterium tuberculosis* and related organisms. Proc. Soc. Exp. Biol. Med..

[45] Maxwell D.N., Perl T.M., Cutrell J.B. (2020). “The art of war” in the era of coronavirus disease 2019 (COVID-19). Clin. Infect. Dis..

[46] Caven B. (1980). The Punic Wars.

[47] Toland J. (1959). Battle: The Story of the Bulge.

